# Management of children with prolonged diarrhea

**DOI:** 10.12688/f1000research.7469.1

**Published:** 2016-02-23

**Authors:** Antonietta Giannattasio, Alfredo Guarino, Andrea Lo Vecchio

**Affiliations:** 1 Department of Translational Medical Sciences - Section of Pediatrics, University of Naples, Federico II, Via S. Pansini 5, Naples, 80131, Italy

**Keywords:** Prolonged Diarrhea, Persistent Diarrhea, Children, Malnutrition

## Abstract

Prolonged diarrhea is usually defined as acute-onset diarrhea lasting 7 days or more, but less than 14 days. Its trend has been declining in recent years because of improvement in the management of acute diarrhea, which represents the ideal strategy to prevent prolonged diarrhea. The pathogenesis of prolonged diarrhea is multifactorial and essentially based on persistent mucosal damage due to specific infections or sequential infections with different pathogens, host-related factors including micronutrient and/or vitamin deficiency, undernutrition and immunodeficiency, high mucosal permeability due to previous infectious processes and nutrient deficiency with consequential malabsorption, and microbiota disruption. Infections seem to play a major role in causing prolonged diarrhea in both developing and developed areas. However, single etiologic pathogens have not been identified, and the pattern of agents varies according to settings, host risk factors, and previous use of antibiotics and other drugs. The management of prolonged diarrhea is complex. Because of the wide etiologic spectrum, diagnostic algorithms should take into consideration the age of the patient, clinical and epidemiological factors, and the nutritional status and should always include a search for enteric pathogens. Often, expensive laboratory evaluations are of little benefit in guiding therapy, and an empirical approach may be effective in the majority of cases. The presence or absence of weight loss is crucial for driving the initial management of prolonged diarrhea. If there is no weight loss, generally there is no need for further evaluation. If weight loss is present, empiric anti-infectious therapy or elimination diet may be considered once specific etiologies have been excluded.

## Introduction and context

Diarrheal disorders are a major health problem in pediatrics worldwide. Accounting for more than 750,000 deaths in children under the age of 5 per year, they are the second leading cause of death in this population according to the World Health Organization (WHO) ^1^. Definitions of diarrheal episodes are usually based on the duration of symptoms rather than etiology. However, there is little consistency and agreement on the definition of acute, prolonged, persistent, and chronic diarrhea in pediatric and adult subjects and in developed and developing countries. In a systematic review on 138 trials, Johnston *et al.* identified 64 different definitions of diarrhea and 69 definitions of diarrhea resolution. The definitions provided by the WHO were the most commonly used ( Table 1) ^2^.

**Table 1.** Definitions of diarrheal illnesses.

**Diarrhea**	The most commonly recognized definition of diarrhea is based on World Health Organization parameters and define diarrhea by the passage of 3 or looser than normal stools in the preceding 24-hour period. An episode of diarrhea is defined as lasting 1 day or more and usually ends after at least 2 days without diarrhea.
**Acute diarrhea**	Episode of self-limiting diarrhea with acute onset, typically lasting 5 to 7 days. In most cases, it is due to an intestinal infection and may be combined to fever and vomiting, meeting the definition of acute gastroenteritis. Acute diarrhea may be also related to extra-intestinal infections (i.e. urinary infection, viral respiratory infections), food-poisoning, iatrogenic intestinal damage (i.e. chemotherapy, radiotherapy) or other intestinal and extra-intestinal diseases such as acute appendicitis.
**Prolonged diarrhea**	Acute onset diarrhea lasting from 7 to 14 days not covering the definition of persistent diarrhea. It is usually due to persistent infections or to post-infectious intestinal damage (i.e. carbohydrate malabsorption, small intestine bacterial overgrowth) that may prolong the duration of diarrhea behind the expected time. Some experts refer to this as acute-protracted diarrhea.
**Persistent diarrhea**	Diarrhea lasting 14 days or more, usually associated with weight loss, ultimately leading to severe nutritional impairment and that may require clinical nutrition. The classical definition of persistent diarrhea was intended to exclude some causes of chronic diarrhea such as celiac disease or inflammatory bowel diseases.
**Chronic diarrhea**	In many contexts chronic diarrhea is a synonymous of persistent diarrhea. The World Health Organization uses this definition rather than persistent diarrhea. However, chronic diarrhea usually does not have an acute onset and is the manifestation of structural and inflammatory bowel disorders. Some experts refer to chronic diarrhea in case of episodes lasting more than 4 weeks.
**Post-infectious diarrhea**	Acute onset diarrhea lasting 7 to 14 days and following an episode of acute gastroenteritis. This definition is covered by prolonged diarrhea.
**Intractable diarrhea**	Non-infectious diarrhea lasting more than 14 days, intractable despite extensive hospital therapy. Typical of young infants, usually below 3 months (but not only). Typically needs intravenous fluids or clinical nutrition and is related to high mortality.
**Congenital diarrhea**	Congenital diarrhea is an inherited enteropathy with a typical onset early in life. For many of these conditions, severe chronic diarrhea represents the main clinical manifestation, while in others, diarrhea is only a component of a more complex multi-organ or systemic disease.

Most diarrheal illnesses last 5–7 days and are due to self-limiting intestinal infections. These episodes are usually defined as acute diarrhea (AD). According to WHO, episodes of diarrhea are usually classified as AD when they last up to a maximum of 14 days and “persistent or chronic diarrhea” when they last >14 days ^3^. However, a subset of children experiences an acute-onset diarrhea lasting 7 days or more, but less than 14 days; this may be defined as prolonged diarrhea (ProD) and usually indicates an episode that continues beyond the expected duration of a typical acute infectious diarrhea ^4^. This entity accounts for a relatively small but consistent number of diarrheal episodes that, especially in developing countries, is associated with a high risk of mortality and morbidity ^4^. In this review, we define ProD as an episode of diarrhea of acute onset lasting 7 to 13 days and persistent diarrhea (PD) as diarrhea lasting 14 or more days.

## Epidemiology and risk factors

Data on the etiology and epidemiology of ProD are limited because of the huge variability in defining diarrheal disorders. Some evidence may be extrapolated from studies reporting data on PD ^5– 7^.

In a study in Israeli children, about a quarter of the study population presented with ProD (lasting 8–13 days) and 14–18% with PD (14 or more days’ duration) ^6^. However, Moore *et al.* firstly proposed the definition of ProD as a specific disorder and reported an incidence of 12% of all diarrheal cases in a large Brazilian cohort, accounting for a quarter of all days of diarrhea recorded in the 10-year study period ^4^. In the same population, less than 5% presented with PD. It should be noted that when a diarrheal episode progresses from acute to ProD, there is a 6-fold higher risk that the episode will evolve into PD ^4^.

ProD is more common in children aged 6 to 24 months and peaks in the second semester of life ^4^. Children who developed ProD in their first year of life have a doubled risk of developing PD at pre-school age ^4^. In addition, children experiencing severe diarrhea and dysenteric illnesses with blood and mucus in their stools are more likely to present with a course longer than those who present with mild-to-moderate diseases ^8, 9^.

These findings, that demonstrate a close relationship between ProD and PD, may be due to different mechanisms: on one hand, ProD affects child growth and mucosal immunity and impacts on gut microflora and intestinal barrier functions; on the other hand, the increased risk of subsequent episodes may be related to specific individual features or to genetic, nutritional, or environmental characteristics that predispose to persistent intestinal illnesses. Mainly in developing areas, ProD is linked with malnutrition in a complex cause-effect relationship, implicating a multifactorial “vicious cycle” involving intestinal infections, microflora disruption, micronutrient deficiency, and immunodeficiency. The role of malnutrition is supported by the evidence that non-breast-fed children and those who are weaned early or recently exposed to formula, as well as children with underlying malnutrition, vitamin deficiency, and wasting, are at increased risk of developing ProD ^4, 8– 10^.

Environmental factors also contribute to ProD, since living in poor areas with poor hygiene conditions and low mothers’ education expose children to a doubled risk of developing ProD ^4^.

Finally, the risk of ProD is reduced by half for 10 years’ increase in maternal age, and if a mother completes primary school, the risk of ProD and PD in her child decreases ^6^.

Irrespective of the etiology and risk factors, children with ProD have a higher risk of nutritional derangement, micronutrient deficiency, risk of developing PD, infections, and immunodeficiency.

## Etiology and pathophysiology

**Figure 1.**
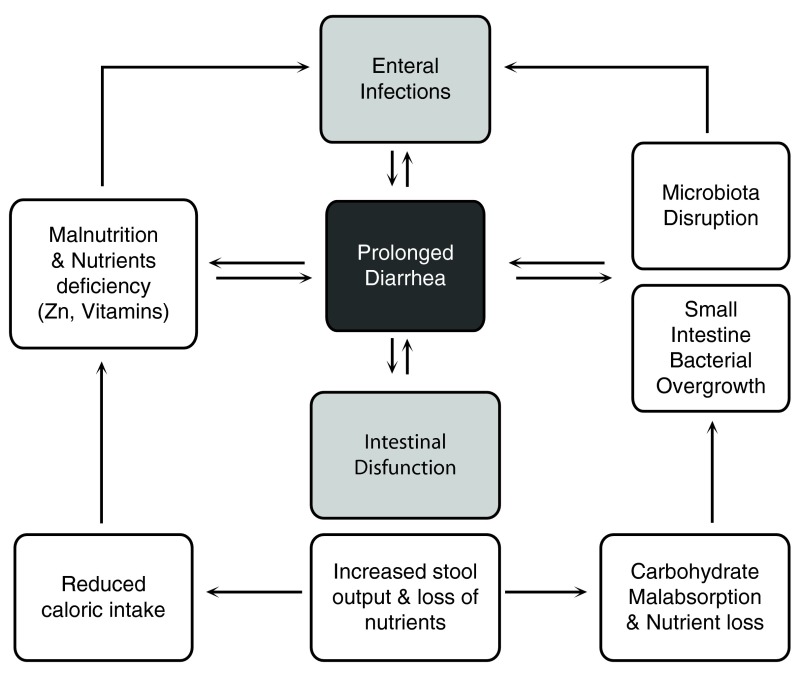
Multifactorial etiology of prolonged diarrhea. “Vicious cycle” of prolonged diarrhea involves intestinal infections, microflora disruption, micronutrient deficit, undernutrition, and immunodeficiency.

The pathogenesis of ProD is multifactorial and essentially based on 1) persistent mucosal damage due to specific agents or sequential infections with different pathogens, 2) host-related factors including micronutrient and/or vitamin deficiency, undernutrition, and immunodeficiency, 3) high mucosal permeability due to previous infectious processes and nutrient deficiency with consequent malabsorption, and 4) microbiota disruption ( Figure 1). In some cases, ProD may represent the onset of chronic intestinal disorders including celiac disease, inflammatory bowel disease (IBD), and autoimmune enteropathies that are usually characterized by PD.

**Table 2.** Etiology of Prolonged Diarrhea in children. HIV: human immunodeficiency virus.

Infections
**Viruses**
Rotavirus Norovirus Sapovirus Astrovirus Cytomegalovirus (HIV-infected children)
Bacteria
Shigella Enteroaggregative E. coli (EAgg EC) Enteropathogenic E. coli (EPEC) Clostridium difficile Campylobacter Yersinia Mycobacterium avium complex (HIV-infected children)
Parasites
Cryptosporidium Giardia lamblia Cyclospora Entamoeba histolytica
Small Intestine Bacterial Overgrowth – SIBO
Underlying malnutrition
Vitamins and Micronutrients deficiency (Zinc, Vitamin A)
Food-induced diarrhea
Lactose intolerance Carbohydrate malabsorption Cow-milk protein intolerance Food allergy Celiac disease
Antibiotic-associated diarrhea

In children with PD, electron microscopy shows shortening of villi, decrease in number and height of microvilli, blunting of borders of enterocytes, loss of glycocalyx, and presence of mucous pseudomembranes coating the epithelial surface. In addition, children with PD often have marked mucosal inflammation when compared to children with AD, presenting higher interferon-gamma response, significant elevation of fecal lactoferrin, interleukin (IL)-8, and IL-1β, and a higher percentage of CD8 ^+^ T cells ^11– 13^. More than half of children with severe AD or ProD develop protein-losing enteropathy ^14^. Also, children with ProD share some of these immunological characteristics.

### Microbial agents

Although infections are a major cause of ProD, there is no clear evidence of a role for selected pathogens in inducing ProD ^6, 8, 14^.

In developing countries, many of the bacterial pathogens responsible for AD and dysentery also cause PD. Invasive diarrhea due to *Shigella* contributes to ProD and PD, and the prevalence of *Shigella* among children with ProD and PD is higher than in those with AD ^6, 8, 15– 17^. Enteropathogenic *Escherichia coli* have been found in at least 25% of children tested in low- and middle-income countries and may cause ProD in some settings ^5, 7, 8^.

Selected viruses may be responsible for ProD. Rotavirus, norovirus, and sapovirus have been found in as many as 50% of diarrheal episodes resolving within the third week in children living in the United States ^18^. In some cases, such as rotavirus infection, ProD could be due to susceptibility to other infections or to nutrient malabsorption rather than being the direct result of the pathogenic agent ^11^. About 10% of astrovirus infections may complicate AD with prolonged episodes of diarrhea, even in non-immunocompromised patients ^19^.

Parasites including *Giardia*, *Cryptosporidium*, and *Cyclospora* have been related to ProD and PD in developing areas. *Cryptosporidium*, a common agent of ProD and PD in HIV-infected children, was frequently isolated also in immunocompetent children ^4^.

Although 35–70% of children with PD tested positive for at least one pathogen, multiple isolations are common, making it difficult to distinguish bystanders from agents causing illness ^5, 6^.

In addition, the pattern of microorganisms that tends to be associated with ProD is different in developed and developing countries. In the former, viruses are more frequently found, whereas in developing countries, specific bacteria and protozoa are more common.

### Small intestinal bacterial overgrowth

A further mechanism that may cause ProD is so-called small intestinal bacterial overgrowth (SIBO). It involves the colonization of the small intestine by bacteria that are usually found in the colonic microbiota or an increase in their number. SIBO is more common in children with underlying intestinal diseases, such as blind loop syndrome, dysmotility, or inflammatory diseases of the intestine. However, SIBO may be a possible complication of AD due to recent infection, the presence in the intestinal lumen of carbohydrates and short-chain fatty acids, the temporary alteration of gut motility, or previous antimicrobial use. It has been associated with antacid therapy. The bacterial overgrowth in the small intestine causes inflammation and malabsorption of liposoluble vitamins and steatorrhea with further worsening of diarrhea and malnutrition.

### Malnutrition and malabsorption

ProD and PD are commonly seen in association with malnutrition and micronutrient deficiencies in developing areas. The latter conditions cause the impairment of immunological mechanisms for clearing infections as well as delayed intestinal repair, thereby contributing to the vicious cycle between diarrhea and malnutrition ^20^.

PD is also due to malabsorption of select nutrients. Food intolerance and food allergy may be responsible for ProD. The mucosal damage secondary to infection leads to loss of lactase activities with a consequent malabsorption of carbohydrates that, due to their osmotic power, worsen and perpetuate diarrhea.

In addition, the increased permeability related to both acute infection and underlying malnutrition may potentially lead to food-antigen sensitization.

### Post-infectious irritable bowel syndrome

Some children and adolescents may develop functional gastrointestinal symptoms, including PD, following a single infectious episode of diarrhea. The clinical picture is characterized by diarrhea and abdominal pain without loss of body weight. This is a syndrome defined as chronic non-specific diarrhea of childhood or “toddler’s diarrhea” and is currently named post-infectious irritable bowel syndrome. The Rome III criteria define the type of symptoms and their duration in age groups ^21^. Based on age-related criteria, the diagnosis can be made without further investigation.

## Diagnostic approach

The management of ProD is complex because the etiology and pathogenesis are complex. Furthermore, the majority of available data on management and treatment are focused on PD rather than on ProD and very few studies have examined ProD as a distinct category ^4^.

**Figure 2.**
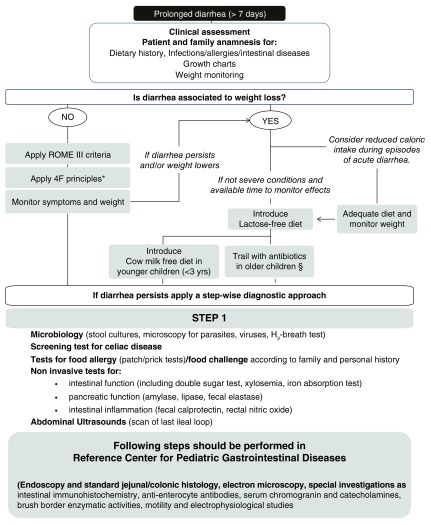
Diagnostic and therapeutic approach to prolonged diarrhea. *Feeding pattern should be normalized according to the “4F” role: fat (increase dietary lipids to at least 35–40% of total daily energy intake), fiber (normalize fiber intake by introduction of fruits and wholegrain bread), fluid (restrict fluid intake if history is significant for high fluid consumption), and fruit juice (discourage overconsumption of fruit juices, especially those containing sorbitol or a high fructose/glucose ratio). § Empiric antibiotic treatment should cover most probable enteric infections ( *Shigella* and enteropathogenic *Escherichia coli*) and/or small intestine bacterial overgrowth.

In developing countries, the main consequences of ProD are nutritional derangement, morbidity (with an increased risk of hospital admission), and even death ^14^. In this setting, it is difficult to perform expensive and time-consuming tests to identify the etiology of ProD. Optimal management of AD is the ideal strategy to prevent ProD. It includes appropriate fluid replacement, zinc treatment, and optimal nutrition in developing countries ^22^. Rotavirus vaccination, promotion of breast-feeding, promotion of hand washing, improved water supply and quality, and community-wide sanitation indirectly prevent ProD ^23, 24^. However, the diagnostic approach to and the treatment of ProD is a challenge. Because of the wide etiologic spectrum, diagnostic algorithms of ProD should also include the age of the patient, clinical and epidemiological factors, and the results of microbiological investigations, when available. An algorithm for ProD is provided in Figure 2. The algorithm is intended as a general tool, and decisions must take into consideration medical history and clinical features of individual cases as described below.

### Clinical evaluation

Specific clues in the family and personal history (previous episodes of AD or ProD, history of chronic diseases or food allergy, and immunological status) may provide useful indications.

Initial clinical examination should include the evaluation of general and nutritional status and the presence of weight loss. Dehydration requires prompt supportive interventions to stabilize the child. Malnutrition may precede the onset of diarrhea, contributing to its duration, or it could be the consequence of the disease. The presence or absence of weight loss may help drive the subsequent diagnostic and therapeutic approach ( Figure 2).

Some children may lose weight as a consequence of poor caloric input after an episode of AD, and in these cases, reintroduction of a free diet is generally effective. However, most children with ProD and weight loss deserve specific medical interventions and careful follow up. If first-line therapeutic interventions do not result in a clear clinical improvement and diarrhea persists, a referral to the gastroenterologist is needed ( Figure 2).

If there is no evidence of weight loss, in most cases there is no need for further investigation. However, a clinical re-evaluation with assessment of the trend of symptoms and weight and revision of nutritional regimens may be considered. Children with persistence of symptoms may need to be referred to the gastroenterologist for further care.

### Laboratory and other investigations

The initial diagnostic workup for children with ProD should include stool cultures and a search for parasites and enteric viruses. However, the wide pattern of microorganisms potentially involved requires high-level techniques.

Although traditional culture is still an invaluable tool in clinical settings, in some instances other techniques are needed for the identification and differentiation of bacterial species ^25^. For other pathogens, it is more important to identify the toxins than the organisms themselves, as in the case of enterotoxigenic *E. coli*, Shiga toxin of enterohemorrhagic *E. coli*, and *Clostridium difficile* ^26^.

Light microscopy represents the traditional technique used to diagnose intestinal parasites. However, its sensitivity depends on the burden of infection, the stage and delivery to the labs of the specimen, and the experience of the observer ^26^. More sensitive and specific enzyme-linked immunosorbent assay (ELISA) and polymerase chain reaction (PCR) analysis are used to detect protozoa in fecal samples, but these assays are still not routinely available.

Enteric viruses are commonly searched for by means of ELISA and latex agglutination analysis. However, molecular genetic techniques detect a wider spectrum of pathogens than conventional techniques and provide information about their molecular epidemiology ^25^. Again, these techniques are not readily available in primary care settings and in resource-limited areas.

A search for SIBO should also be included in the diagnostic work-up of ProD. Breath hydrogen test may identify an abnormal bacterial proliferation in the small bowel; it can also be used to detect carbohydrate malabsorption ^27^. However, the sensitivity and specificity of this test is low and it is difficult to perform, mainly in young children. Alternatively, a simple measurement of stool pH or a test for reducing substances (Clinitest) may be easily done at the bedside to investigate carbohydrate (usually lactose) malabsorption. Other specific diagnostic tests, including evaluation of intestinal function and inflammation, imaging, and endoscopy, should be performed in case of persistence of diarrhea in the setting of pediatric gastroenterology.

Non-invasive diagnostic tests for intestinal and pancreatic function (including dual absorption test, xylosemia, iron oral load, lipase, and fecal elastase) and inflammation (fecal calprotectin and last ileal loop abdominal ultrasound) may provide useful information for diagnosis, when available ^27, 28^. Although abdominal ultrasound may be affected by subjective evaluation if an expert consultant is available, it may be of support in management. The association of a negative scan of last ileal loop with negative fecal calprotectin significantly reduces the possibility of IBD ^28^. The use of noninvasive diagnostic tests in the diagnostic algorithm may reduce invasive procedures. However, if diagnosis is not obtained otherwise, endoscopy with biopsy should be considered.

In young children with a familial history of atopy and suggestive personal history and clinical features, skin tests for the screening of food allergy (including patch tests and prick tests) may be performed and elimination diet with subsequent challenge should be considered ^29^.

## Treatment

### Empiric treatment

Expensive laboratory evaluations usually are of little benefit in guiding the successful treatment of ProD, and an empirical therapeutic approach may be effective in the majority of cases ( Figure 2).

If there is no weight loss, the approach should be conservative and no investigations are generally needed. A review of the diet is worthwhile, including a check on a possible excess of sugar-containing drinks as a cause of diarrhea. Feeding pattern should be normalized according to the “4F” rule: fat (increase dietary lipids to at least 35–40% of total daily energy intake), fiber (normalize fiber intake by introduction of fruits and wholegrain bread), fluid (restrict fluid intake if history is significant for high fluid consumption), and fruit juice (discourage overconsumption of fruit juices, especially those containing sorbitol or with a high fructose/glucose ratio).

A lactose-free diet can be started if weight loss is present. Some children with ProD who do not have weight loss at first presentation may benefit from a lactose-free diet since disaccharidase deficiency secondary to AD is relatively common. Exclusion diets are usually administered with the double purpose of overcoming food intolerance, which may be the primary cause of ProD, or its complication. The sequence of elimination should be graded from less (e.g. cow’s milk protein hydrolysate) to more restricted diets (amino acid-based formula) according to the child’s clinical conditions. This approach should be reserved for infants and young children.

### Antimicrobials

ProD is often an infection-induced illness in the majority of cases ^30^. However, considering that pathogens associated with ProD are also often found in healthy children without diarrhea ^7^, even when an enteric pathogen is detected, it is not always clear that this is the cause of the illness.

Antimicrobial agents are indicated for the treatment of selected parasites ^31^ and selected enteropathogenic bacteria, such as enteropathogenic *E. coli* and enteroaggregative *E. coli* ^14^. Pathogens such as *Shigella* and *Cryptosporidium* are commonly associated with ProD in tropical, developing countries and should be treated in case of ProD ^4^.

Nitazoxanide is a broad-spectrum antimicrobial agent with activity against protozoa, nematodes, cestodes, trematodes, and bacteria, with a favorable safety profile ^32, 33^. It is effective in childhood cryptosporidiosis ^34^ but not consistently in undernourished children or in HIV-infected patients ^35^. Anecdotal cases of children successfully treated with nitazoxanide because of PD (<30 days) have been reported ^36^. This strategy seems to be effective in select situations, saving time-consuming tests to identify the cause of diarrhea ^36^.

Metronidazole can be used for *Giardia*, and trimethoprim-sulfamethoxazole can be used for *Cyclospora* and as a second-line antibacterial drug for a number of pathogens ^14^.

Very few data on the treatment of viral ProD are available. Human immunoglobulin, available for intravenous use, may be administered orally (300 mg/kg of body weight) in a single dose. The rationale of passive immunotherapy is based on the demonstration of neutralizing antibodies against all viruses in a medical preparation of immunoglobulins ^37^. They are found in the stools after administration, and this treatment reduces the duration of stay in severe and/or immunocompromised patients with AD and in patients with severe diarrheal episodes due to rotavirus ^38, 39^. This treatment seems to also have a potential role in immunocompromised patients with norovirus enteritis ^40^. In this population, a positive trend towards resolution of diarrhea and decreased stool output in the treatment group compared with placebo was found, but no benefit was reported for length of hospital stay or hospital cost ^40^. However, no data on oral immunoglobulins and viral-ProD is available, with the exception of rotavirus ^41^.

Ciprofloxacin is effective in cases of diarrhea associated with enteroaggregative *E. coli* in HIV-infected adults ^42^. However, its efficacy needs to be proven in a large-scale trial in children with ProD.

The efficacy of antimicrobials in children with ProD in whom etiology is unknown is even more controversial. In a recent systematic review in young children with PD of unknown or non-specific cause from developing countries, no difference was demonstrated for oral gentamicin or metronidazole compared with placebo, whereas sulfamethoxazole-trimethoprim was more effective than placebo for diarrhea at 7 days and for total stool volume ^43^. To date, the evidence to recommend the use of antibiotics in ProD of unknown or non-specific causes is still limited.

### Probiotics

Some data support the use of probiotics in ProD. *Lactobacillus* spp. and *Saccharomyces boulardii* significantly reduced the number of stools and duration of diarrhea in children with PD ^44^.

A recent Cochrane review showed that probiotics shortened the duration of diarrhea and reduced stool frequency and hospital stay. However, only four trials with a small number of participants were available for meta-analysis. The authors concluded that, although probiotics appear to hold promise as adjunctive therapy, there is insufficient evidence to recommend their routine use in children with ProD ^45^.

### Nutritional interventions

Dietary treatment is crucial in ProD. However, nutritional interventions are often expensive and poorly applicable in developing countries. Locally available, inexpensive foods and vitamin and mineral supplementation have been proposed ^46– 48^. Locally tailored nutritional interventions are ideal in the developing world: they are inexpensive and culturally acceptable, provide sufficient levels of energy and nutrients to malnourished children, and allow the continuation of dietary therapy at home ^46, 49^.

A multicenter study in severely ill children aged 4–36 months with PD showed a high success rate of a dietary regimen using inexpensive, locally available foods (variable association of rice, maize, lentils, chicken, yoghurt, milk, sucrose or glucose, and oil) and vitamin and mineral supplementation ^46^. Although a recent systematic review underlined the low-quality evidence of these studies, the authors found no evidence to support the use of proprietary formulas or specialized ingredients over the use of locally produced and readily available foods in the treatment of either AD or PD ^50^.

Other approaches that have been evaluated include the use of amylase-rich flours in cereal-based porridges to decrease viscosity and thus increase nutrient density and children’s nutrient intake. However, these trials have been performed only in children with AD ^51^. Also, mixed diets including specific ingredients thought or known to have antidiarrheal properties, such as green banana, have been tested. These reports showed a significantly higher cumulative probability of recovery in children ^52, 53^.

Children with ProD and malnutrition may present with deficiency of selected micronutrients such as vitamin A, zinc, folic acid, copper, and selenium ^54^. Zinc was found to have therapeutic efficacy (typically resolution of small bowel damage and shortening duration of diarrhea) in several trials of PD ^55^. Zinc and vitamin A in combination seem to be even more effective than either vitamin A or zinc alone in reducing PD in developing countries ^56^.

## Conclusion

In children with severe malnutrition, ProD may be the direct result of secondary immunodeficiency and the consequence of a reduction of intestinal absorptive-digestive surface. In this condition, nutritional interventions (nutritional regimen review/optimization and micronutrient supplementation) should be considered with the aim of optimizing the use of residual functioning intestine over time. Enteral nutrition given continuously by feeding tube may be used in severe cases of reduction of intestinal functional surface. However, anti-infectious therapy should also be considered.

Overall, the treatment of ProD is a balance between a possible infectious etiology and a nutritional approach. When, however, the features of irritable bowel syndrome (defined according to the Rome III criteria) are met, no specific medication should be prescribed. However, parental reassurance and diet tailoring (e.g. an increase in fiber intake) may be helpful, and counseling/behavioral therapy may be indicated to reduce anxiety that may trigger gastrointestinal symptoms. If a specific cause of diarrhea is detected, such as celiac disease or IBD, an appropriate therapy should be started.

## Abbreviations

WHO, World Health Organization; AD, acute diarrhea; ProD, prolonged diarrhea; PD, persistent diarrhea; IBD, inflammatory bowel disease; SIBO, small intestinal bacterial overgrowth.
